# Regional Cerebral Blood Flow Abnormalities in Neurosyphilis: A Pilot SPECT Study

**DOI:** 10.3389/fneur.2021.726006

**Published:** 2021-11-10

**Authors:** Jooyeon J. Im, Hyeonseok Jeong, Young Do Kim, Kyung-Sool Jang, In-Uk Song, Yong-An Chung

**Affiliations:** ^1^Department of Nuclear Medicine, Incheon St. Mary's Hospital, College of Medicine, The Catholic University of Korea, Seoul, South Korea; ^2^Department of Radiology, Incheon St. Mary's Hospital, College of Medicine, The Catholic University of Korea, Seoul, South Korea; ^3^Department of Neurology, Incheon St. Mary's Hospital, College of Medicine, The Catholic University of Korea, Seoul, South Korea; ^4^Department of Neurosurgery, Incheon St. Mary's Hospital, College of Medicine, The Catholic University of Korea, Seoul, South Korea

**Keywords:** neurosyphilis, single-photon emission computed tomography, regional cerebral blood flow, cognitive function, neuroimaging

## Abstract

**Objective:** Clinical and radiological findings on neurosyphilis are fairly non-specific and there is a paucity of functional neuroimaging studies on neurosyphilis other than case reports and case series. The purpose of this study was to investigate brain perfusion abnormalities in patients with neurosyphilis.

**Methods:** Four HIV-negative neurosyphilis patients and 4 healthy controls underwent clinical evaluation, brain technetium-99m ethyl cysteinate dimer (99mTc-ECD) single-photon emission computed tomography (SPECT) imaging, and neuropsychological assessments which included the Mini-Mental State Examination (MMSE), Clinical Dementia Rating (CDR), Clinical Dementia Rating—Sum of Boxes (CDR-SOB), and Global Deterioration Scale (GDS). Voxel-wise differences in regional cerebral blood flow were compared between the two groups.

**Results:** Neuropsychological test results indicated cognitive impairment in all patients. SPECT analysis revealed multifocal hypoperfusion predominantly in the frontal, insular, and posterior cingulate regions in neurosyphilis patients compared with healthy controls (family-wise error corrected *p* < 0.05).

**Conclusions:** Together with previous findings, our results suggest that the hypoperfusion in the frontal, insular, and posterior cingulate regions may reflect cognitive impairments observed in neurosyphilis patients. Further studies with larger samples are needed to confirm our findings.

## Introduction

Neurosyphilis is an infection of the central nervous system by Treponema pallidum (1). Neurosyphilis has been reported to develop in up to 5–10% of patients with untreated syphilis and can occur at any stage of syphilis, which is divided into primary, secondary, latent, and tertiary syphilis (1, 2). The incidence of syphilis has varied over time with a marked decrease after the introduction of penicillin and a surge in the era of HIV/AIDS pandemic followed by another decline due to engaging in safe sexual practices. However, the rates of syphilis began to increase again since 2000 (3). Despite the relative success in controlling syphilis in the post-penicillin era, syphilis remains an important public health issue with an estimated annual global incidence of 12 million (4).

Neurosyphilis is classified into early forms of neurosyphilis which includes asymptomatic neurosyphilis, syphilitic meningitis, and syphilitic meningovasculitis and late forms of neurosyphilis which includes general paresis and tabes dorsalis (5). Early forms of neurosyphilis primarily affects the meninges, cerebrospinal fluid (CSF), and vasculature, and late forms of neurosyphilis affects the brain and spinal cord parenchyma. The clinical manifestations of symptomatic neurosyphilis are diverse and usually non-specific including meningitis, stroke, seizures, headache, hearing loss, vision loss, personality changes, cognitive decline, dementia, or sensory and gait abnormalities (5, 6). The diagnosis of symptomatic neurosyphilis is based on the serological testing, CSF examination, and clinical findings (5). However, diagnosis of neurosyphilis is often difficult because many patients present either non-specific symptoms or are asymptomatic.

Identifying radiological features of neurosyphilis may be useful in aiding the diagnosis and distinguishing the disease from other conditions. Previous neuroimaging studies have reported generalized cerebral atrophy, non-specific white matter lesions, meningeal and CSF enhancement, and signal changes in the frontotemporal lobes as well as some normal findings in neurosyphilis patients (7–9). However, apart from case reports and case series, no prospective studies have been published investigating the cerebral perfusion in neurosyphilis patients. The purpose of this study was to investigate the differences in regional cerebral blood flow (rCBF) between neurosyphilis patients and healthy controls using single-photon emission computed tomography (SPECT).

## Methods

### Participants

Neurosyphilis patients and healthy controls were recruited at the Incheon St. Mary's Hospital (Incheon, South Korea). The diagnosis of neurosyphilis is based on a CSF WBC count of 5 cells/microL or more, CSF protein 45 mg/dl or more and a reactive CSF VDRL. All patients in this study showed a reactive serum FTA-ABS and cognitive impairment including neuropsychiatric symptoms. All patients were HIV-negative. This study excluded patients with following past history: head trauma, existing dementia diagnosis, stroke, brain tumor, epilepsy, sexually transmitted diseases except for syphilis, and other neurological or psychiatric disorders. Healthy controls were age- and sex-matched volunteers who met the same exclusion criteria and inclusion criteria, except for the presence of neurosyphilis. The study was approved by the Institutional Review Board of the Incheon St. Mary's Hospital, and all participants provided written consent form.

### Clinical Assessment

Screening tests included a medical history interview, physical and neurological examinations by a neurologist, routine blood biochemistry and blood count, 12-lead electrocardiogram, chest x-ray, and CSF study including counts of WBC and RBC, protein, glucose, and CSF VDRL. We performed brain magnetic resonance imaging, SPECT scans, and neuropsychological tests before medical treatment. Neuropsychological assessments consisted of the Mini-Mental State Examination (MMSE) (10), Clinical Dementia Rating (CDR) (11), Clinical Dementia Rating—Sum of Boxes (CDR-SOB) (11), and Global Deterioration Scale (GDS) (12).

### Brain SPECT Imaging

Brain SPECT scans were performed using a dual-headed gamma camera (Discovery NM630; GE Healthcare, Milwaukee, WI, USA) equipped with a low-energy fan-beam collimator. Patients were injected with 555–740 MBq of technetium-99m ethyl cysteinate dimer (99mTc-ECD) and rested for ~40 min prior to scanning. Images were taken by rotating the camera a total of 720° at 6-degree intervals at a rate of 12 s per frame (average counts = 1.50 kcts/s). Images were corrected for attenuation using a standard commercial correction routine provided by the scanner vendor and reconstructed into a 128 × 128 matrix with a pixel size of 1.95 × 1.95 × 2.08 mm and a 20% symmetric energy window at 140 keV using the ordered-subset expectation maximization (OSEM) algorithm (6 iterations and 10 subsets) and a Butterworth filter (cut-off frequency of 0.5 cycles/pixel and power of 10.0) to reduce noise.

All SPECT images were pre-processed and analyzed using Statistical Parametric Mapping 12 (SPM; Wellcome Center for Human Neuroimaging, London, UK) implemented in MATLAB R2017b (MathWorks, Natick, MA, USA). The images were spatially normalized to the standard SPECT template provided by SPM. After spatial normalization, the count of each voxel was standardized to the mean voxel count of the whole brain using proportional scaling. The normalized images were then smoothed using a 16-mm full-width half-maximum Gaussian kernel.

A two-sample *t*-test was performed to compare the rCBF between the groups. The voxel-wise significance threshold was set at *p* < 0.001 with a cluster-level family-wise error (FWE) correction at *p* < 0.05.

To assess individual patterns of abnormalities in rCBF, separate two-sample *t*-tests were conducted to compare each patient with the control group. The voxel-level threshold of *p* < 0.005 with cluster-level FWE corrected *p* < 0.05 was applied for the individual analysis.

### Statistical Analysis

Mann-Whitney *U* test was used to compare age between the groups. The significance level was set at *p* < 0.05 (two-tailed). All statistical analyses were performed using Stata/MP 16.0 (Stata Corp., College Station, TX, USA).

## Results

### Demographic and Clinical Characteristics

Four patients with neurosyphilis and four healthy controls were enrolled in the study. Demographic and clinical characteristics are summarized in Table 1. All participants were male, and age did not significantly differ between the neurosyphilis (mean ± standard deviation = 46.3 ± 7.6) and control groups (46.3 ± 6.3, *p* = 0.77). Scores of MMSE, CDR, CDR-SOB, GDS indicated cognitive impairment in patients with neurosyphilis.

**Table 1:** Demographic and clinical characteristics of the participants.

**Participant**	**Age**	**Sex**	**MMSE**	**CDR**	**CDR-SOB**	**GDS**
Patient 1	43	Male	20	1	4.5	4
Patient 2	37	Male	28	0.5	0.5	3
Patient 3	53	Male	22	1	4.5	4
Patient 4	52	Male	23	1	6	5
Control 1	48	Male	30	0	0	1
Control 2	54	Male	28	0	0	1
Control 3	44	Male	30	0	0	1
Control 4	39	Male	29	0	0	1

*CDR, Clinical Dementia Rating; CDR-SOB, Clinical Dementia Rating—Sum of Boxes; GDS, Global Deterioration Scale; MMSE, Mini-Mental State Examination*.

### Brain SPECT Imaging Results

**Figure 1:**
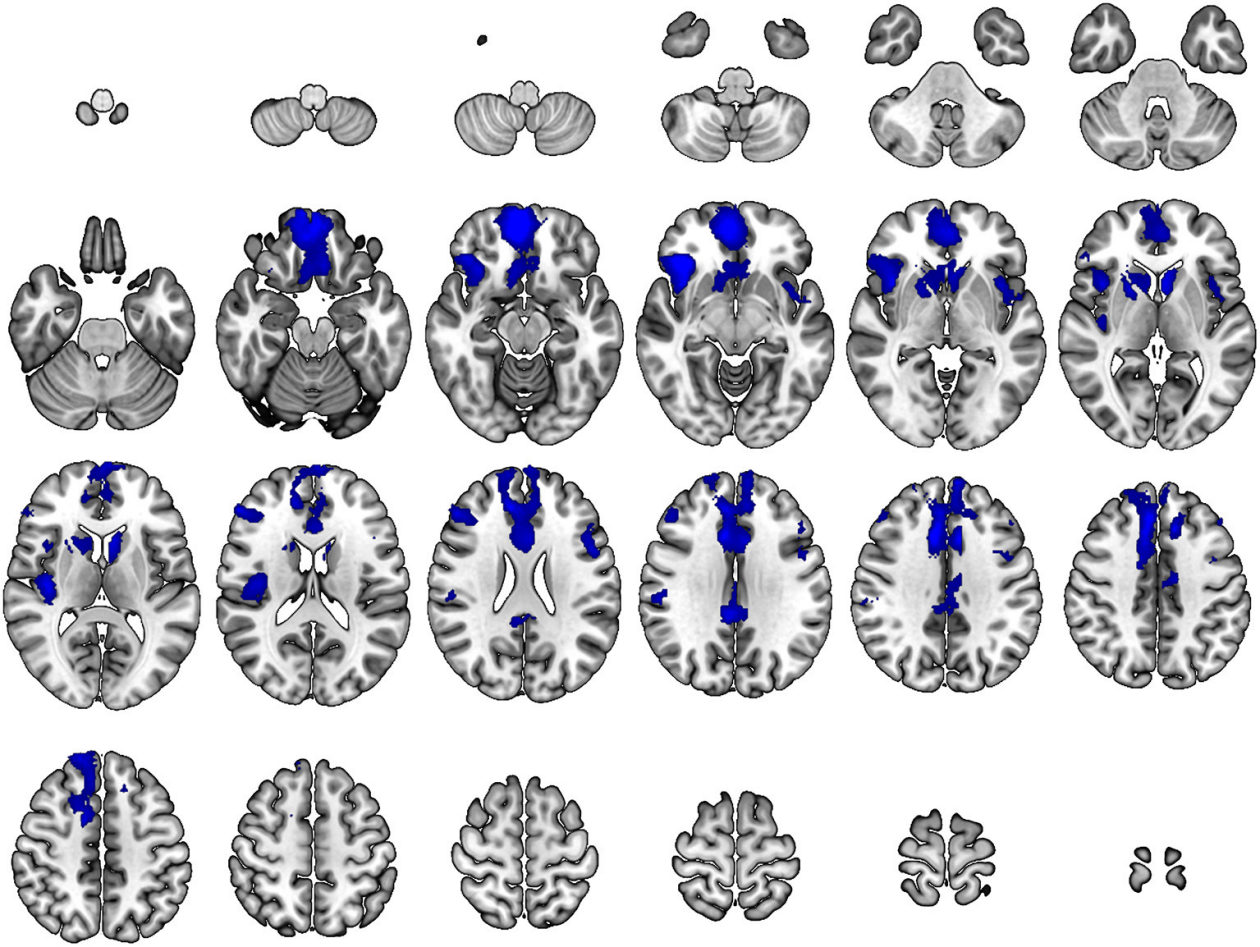
Differences in regional cerebral blood flow between patients with neurosyphilis and healthy controls. At each voxel, decreases in brain perfusion in neurosyphilis patients compared with healthy controls appear in blue. The height threshold is *p* < 0.001 with a cluster-level family-wise error correction at *p* < 0.05. Images are shown in radiological convention.

As compared with controls, neurosyphilis patients showed reduced rCBF in six clusters including the (1) right inferior/middle frontal gyrus and anterior insula (*z* = 4.89, *p* < 0.001), (2) right medial frontal cortex and middle cingulate gyrus (*z* = 4.65, *p* < 0.001), (3) right central operculum and posterior insula (*z* = 4.09, *p* < 0.001), (4) right posterior cingulate gyrus (*z* = 4.08, *p* < 0.001), (5) left anterior insula and superior temporal gyrus (*z* = 3.87, *p* < 0.001), and (6) left inferior/middle frontal gyrus (*z* = 3.74, *p* < 0.001) (Table 2 and Figure 1). No significant clusters of increased rCBF were detected.

**Table 2:** Differences in regional cerebral blood flow between patients with neurosyphilis and healthy controls.

**Region**	***z***	***p***	Coordinates^*^ (x, y, z)	**Cluster size (voxels)**
**Neurosyphilis group** **<** **control group**				
R inferior/middle frontal gyrus, anterior insula	4.89	<0.001	40, 28, −8	1,489
R medial frontal cortex, middle cingulate gyrus	4.65	<0.001	2, 58, −14	8,453
R central operculum, posterior insula	4.09	<0.001	40, −16, 14	669
R posterior cingulate gyrus	4.08	<0.001	4, −36, 28	533
L anterior insula, superior temporal gyrus	3.87	<0.001	−40, 12, 0	302
L inferior/middle frontal gyrus	3.74	<0.001	−50, 10, 26	313
**Neurosyphilis group** **>** **control group**				
None				

* *The coordinates refer to the Montreal Neurological Institute coordinate system. L, left; R, right*.

For each patient, the SPECT images and the results of individual analysis are demonstrated in Supplementary Figures 1–4. Despite some differences of rCBF patterns among patients, frontal and insular hypoperfusion was observed in all patients.

## Discussion

In the current study, HIV-negative neurosyphilis patients showed multifocal hypoperfusion predominantly in the frontal, insular, and posterior cingulate regions compared to healthy controls. Although diffuse hypoperfusion has been found in various central nervous system infections and neurotoxicity such as HIV and alcohol-related dementia (13, 14), the regional pattern of our findings may be associated with cognitive decline found in patients with neurosyphilis.

Our results are in agreement with previous SPECT findings of decreased rCBF in the frontal regions in patients with general paresis (15–18), which is the most common form of neurosyphilis presenting with cognitive impairment and psychiatric symptoms (19). These cases also reported decreased rCBF in the temporal regions (15–18), which was less prominent in our study. In contrast, there are single-case reports that showed increased rCBF in the temporal lobe (20) and frontal and temporo-occipital regions (21). Moreover, in a SPECT study which included 32 patients with early syphilis, a general and patchy hypoperfusion was found in the frontal, temporal, parietal, occipital lobes as well as the basal ganglia, cerebellum, and nerve nucleus (22).

These inconsistencies may be partially attributed to differences in disease stage at diagnosis as hyperperfusion may reflect inflammatory changes in the early phase and hypoperfusion may reflect neural death and decreased metabolism in the late phase (21). Besides that, the uncontrolled nature of case reports and bias from visual inspection might have contributed to the discrepancies as well. In spite of these differences, previous reports consistently showed improved rCBF after successful treatment, suggesting that SPECT may be a useful method in detecting subtle changes in neurosyphilis patients receiving antisyphilitic treatment (15–17, 20, 21).

In the present study, the neurosyphilis patients demonstrated hypoperfusion in multiple frontal regions, particularly in the medial frontal regions. Numerous studies have been published on the role of the medial frontal cortex in a wide range of high-level cognition and its involvement in neurological and psychiatric disorders (23). The medial frontal cortex has been implicated in performance monitoring, motivation, decision making, and social cognition (24–27). Moreover, it has been proposed that the medial frontal cortex is part of a dual hierarchical system for the prefrontal executive function that extends from the posterior to anterior regions in the medial frontal cortex and lateral frontal cortex (26).

We also found hypoperfusion in the insula and posterior cingulate gyrus. In addition to its well-established role in processing interoceptive information, growing evidence suggests that the insula is also involved in cognitive and emotional processing (28). The insula has bidirectional connections with the critical brain areas for cognition such as the orbitofrontal cortex and anterior cingulate cortex, and impairment in the insula has been linked with cognitive impairment across all neuropsychological domains (28). Abnormalities in the posterior cingulate cortex are often associated with impairments in cognitive functions including memory and attention (29). Notably, atrophy and reduced metabolism in the posterior cingulate cortex are seen in Alzheimer's disease (29).

## Conclusion

In conclusion, the present study found the perfusion abnormalities in the frontal, insular, and posterior cingulate regions among HIV-negative neurosyphilis patients. These results, together with previous findings, suggest that the hypoperfusion in these areas may reflect cognitive impairments observed in neurosyphilis patients. Further studies with larger samples are needed to confirm our findings. In addition, multi-modal neuroimaging studies may be useful to evaluate potential associations between structural and functional deficits in these patients.

## Data Availability Statement

The datasets presented in this article are not readily available because the IRB has restrictions on sharing datasets. Requests to access the datasets should be directed to Yong-An Chung, yongan@catholic.ac.kr.

## Ethics Statement

The studies involving human participants were reviewed and approved by Institutional Review Board of the Incheon St. Mary's Hospital. The patients/participants provided their written informed consent to participate in this study.

## Author Contributions

JI and HJ contributed to analysis, interpretation of data, and original draft preparation. YK and K-SJ contributed to data acquisition. I-US and Y-AC contributed to study concept, design, and interpretation of data. All authors contributed to the article and approved the submitted version.

## Funding

This research was supported by the Korea Health Industry Development Institute (KHIDI) and Korea Dementia Research Center (KDRC) (HU21C0081) and the National Research Foundation of Korea (NRF) funded by the Korean government (2020R1C1C1007254).

## Conflict of Interest

The authors declare that the research was conducted in the absence of any commercial or financial relationships that could be construed as a potential conflict of interest.

## Publisher's Note

All claims expressed in this article are solely those of the authors and do not necessarily represent those of their affiliated organizations, or those of the publisher, the editors and the reviewers. Any product that may be evaluated in this article, or claim that may be made by its manufacturer, is not guaranteed or endorsed by the publisher.
