# Supplementary material for: Regional Cerebral Blood Flow Abnormalities in Neurosyphilis: A Pilot SPECT Study
Source: Front Neurol. 2021 Nov 10;12:726006. doi: 10.3389/fneur.2021.726006 (PMC8631505; doi:10.3389/fneur.2021.726006)
Supplement: Supplementary file 1 [file Data_Sheet_1.DOCX]

**Supplement for**

**Regional Cerebral Blood Flow Abnormalities in Neurosyphilis: A Pilot SPECT Study**

**Jooyeon J. Im, Hyeonseok Jeong, Young Do Kim, Kyung-Sool Jang,**

**In-Uk Song*, Yong-An Chung***

* Correspondence:

In-Uk Song (siuy@catholic.ac.kr)

Yong-An Chung (yongan@catholic.ac.kr)

| 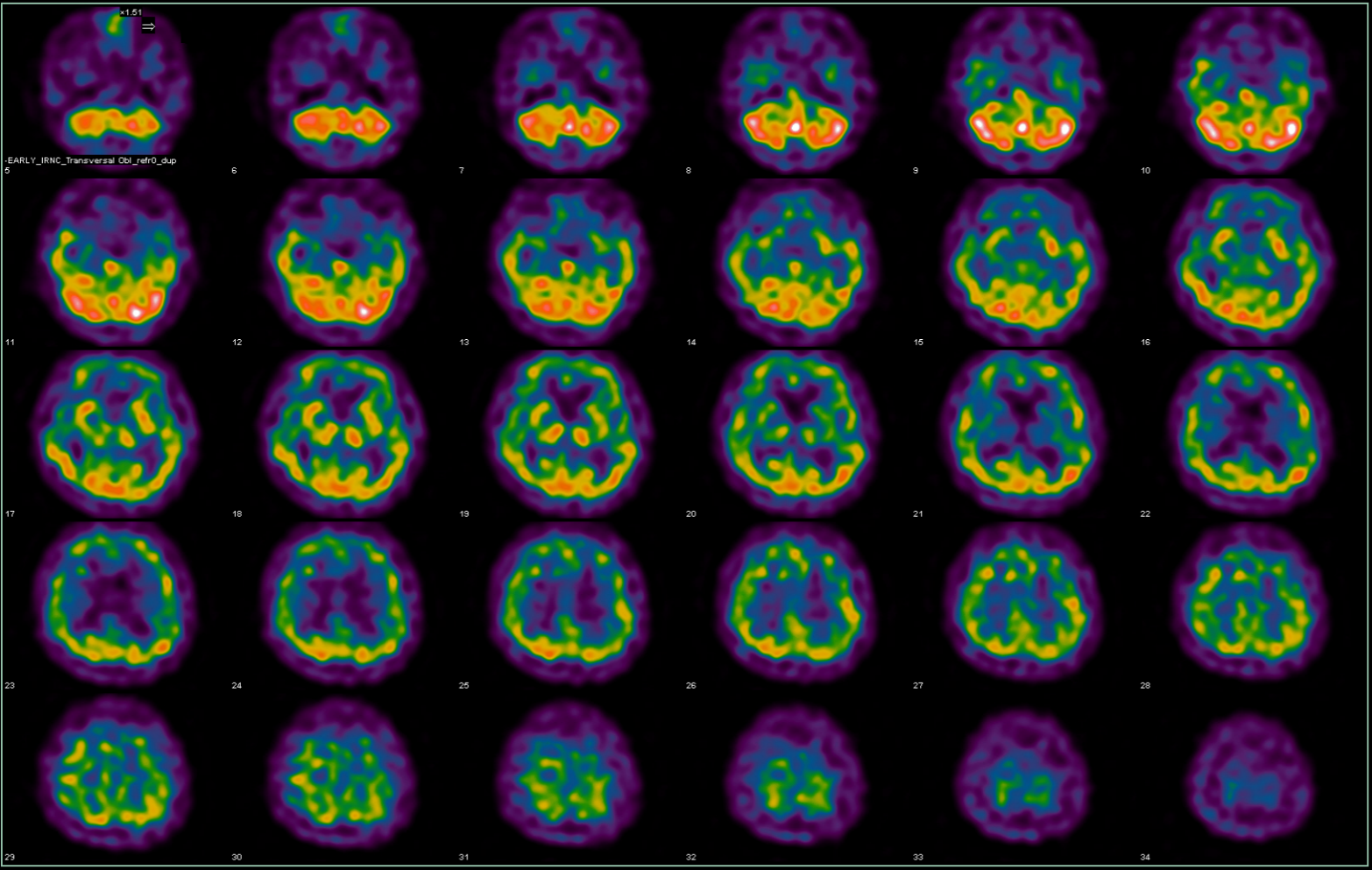  **(A)** |
| --- |
| 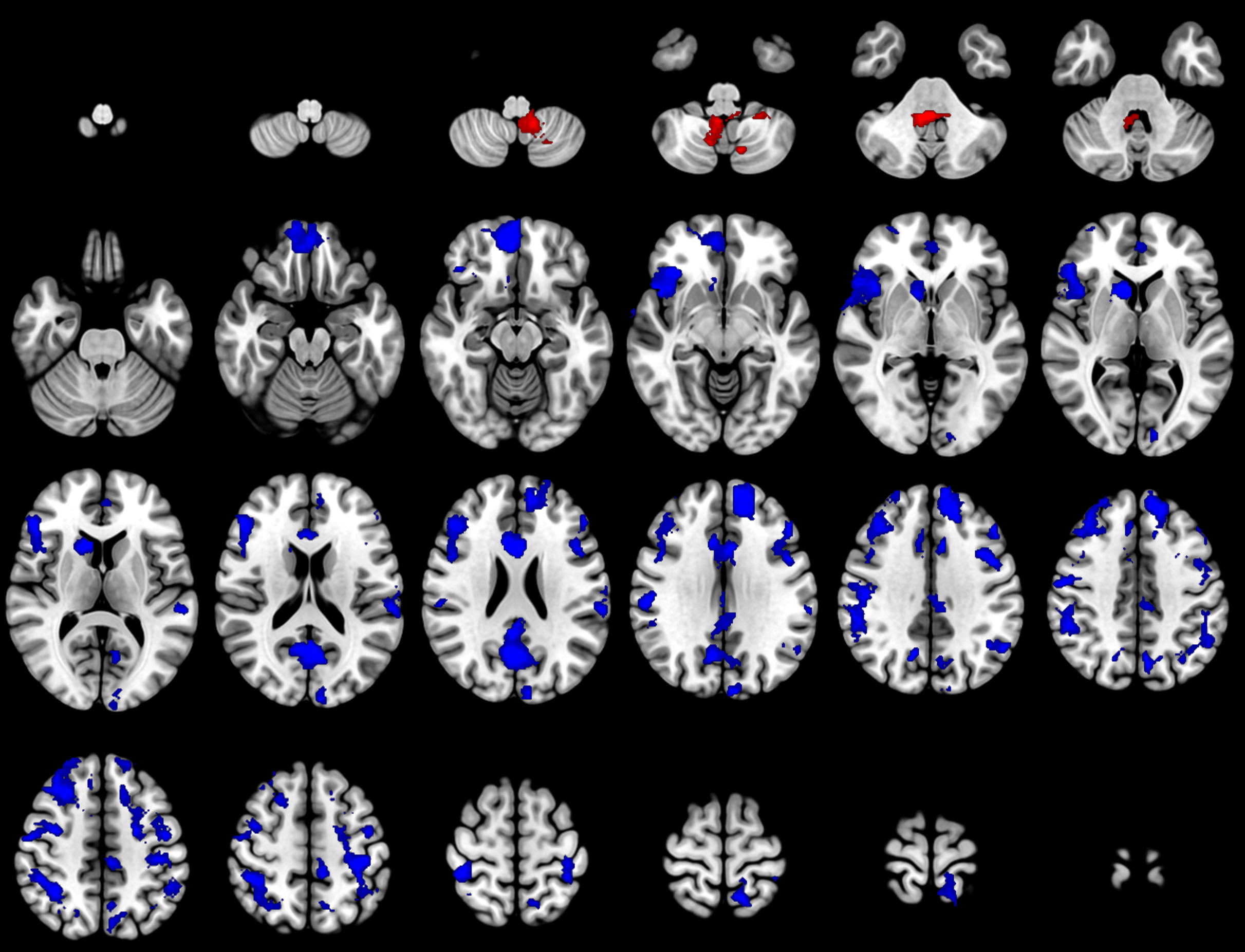  **(B)** |

**Supplementary Figure 1.** (A) SPECT images of Patient 1. (B) Higher (red) or lower (blue) regional cerebral blood flow of Patient 1 compared to the control group. Images are shown in radiological convention.

| 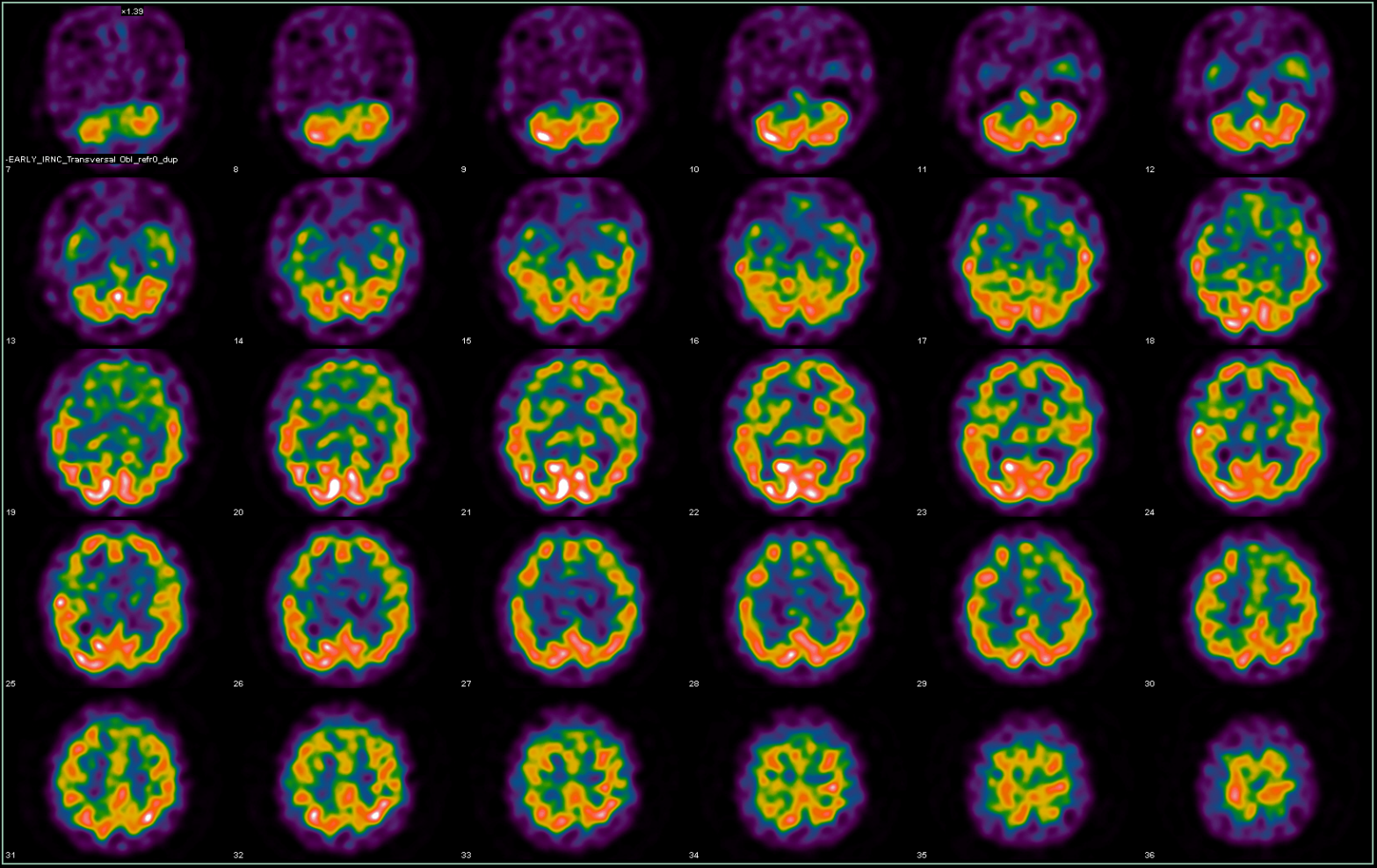  **(A)** |
| --- |
| 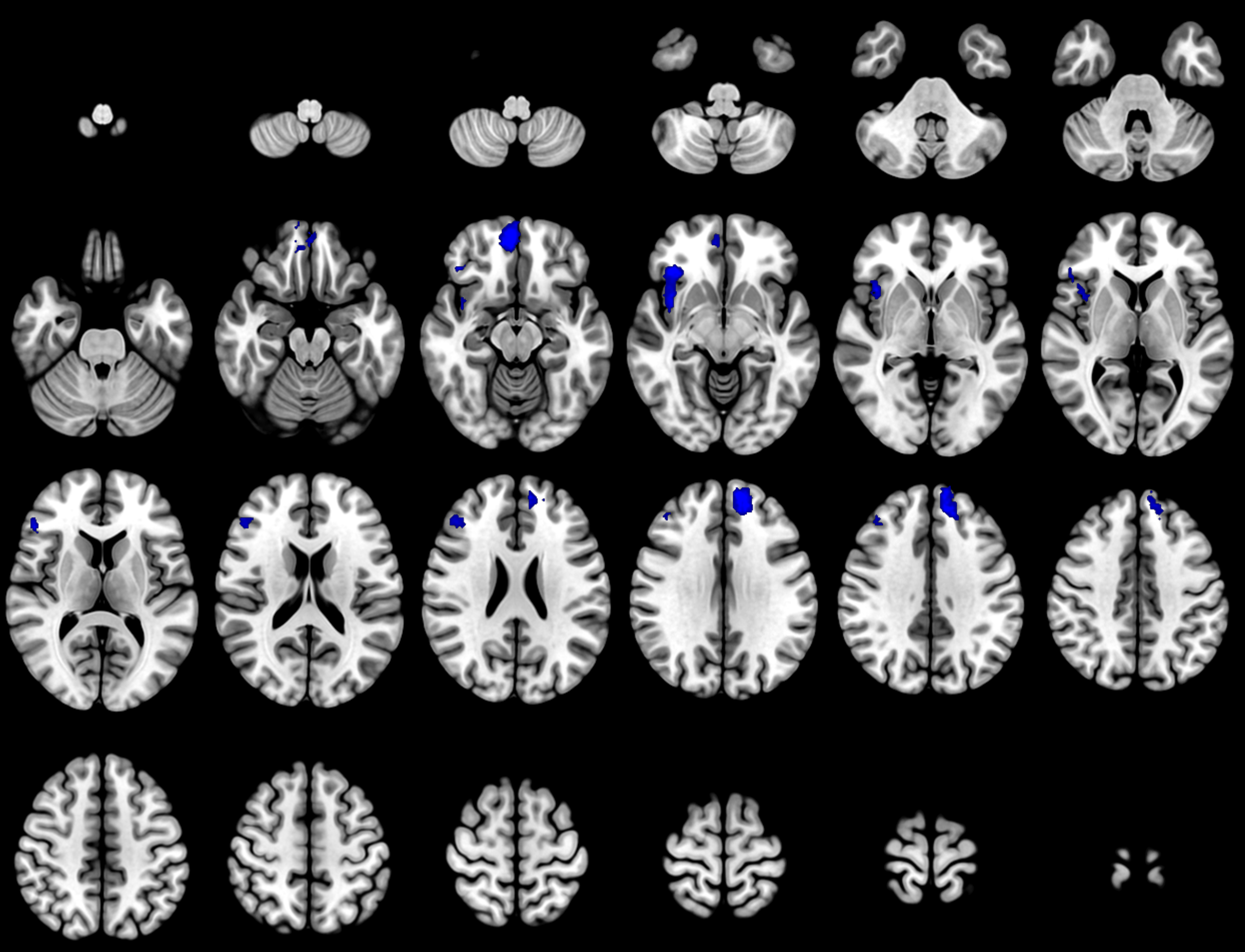  **(B)** |

**Supplementary Figure 2.** (A) SPECT images of Patient 2. (B) Higher (red) or lower (blue) regional cerebral blood flow of Patient 2 compared to the control group. Images are shown in radiological convention.

| 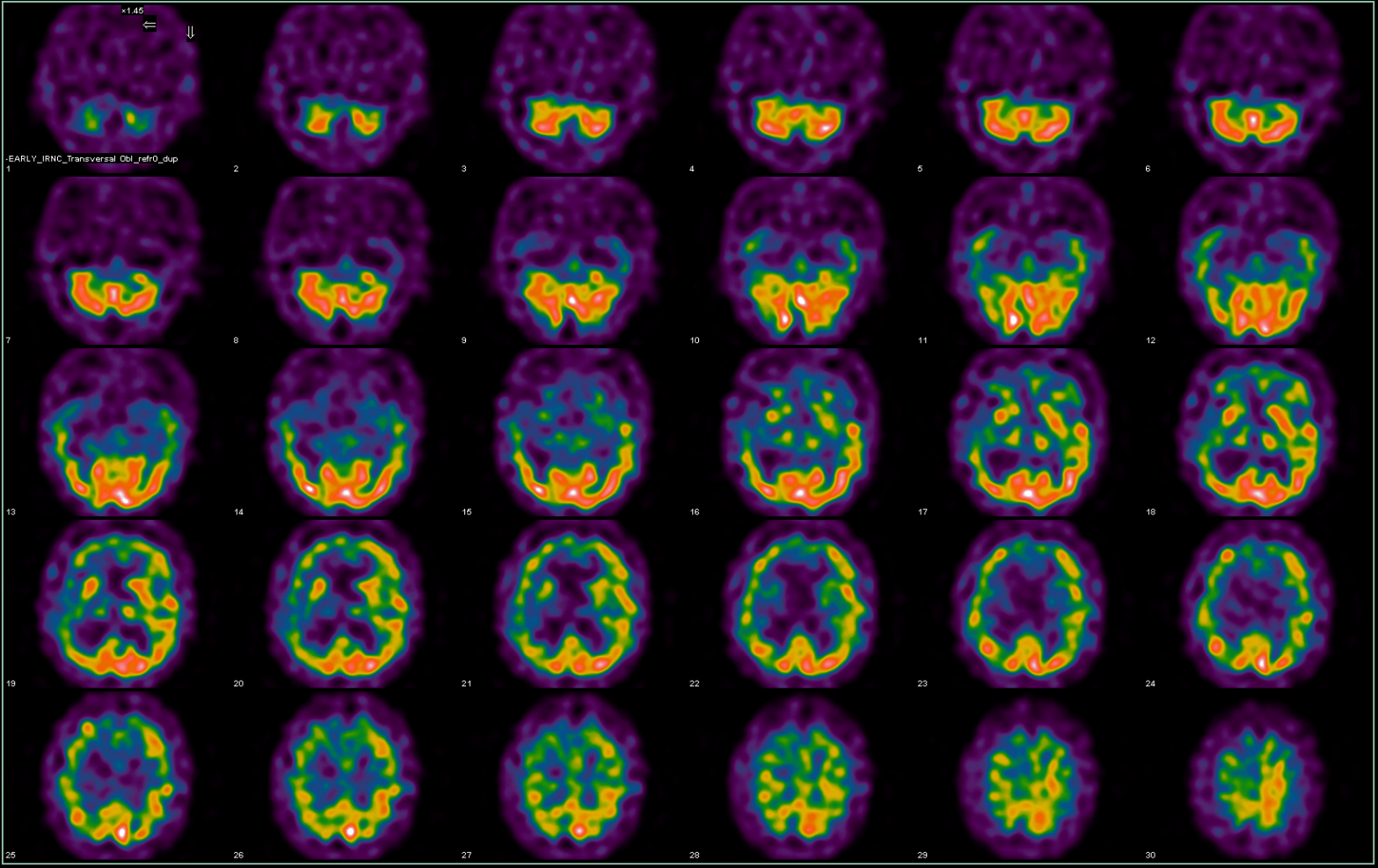  **(A)** |
| --- |
| 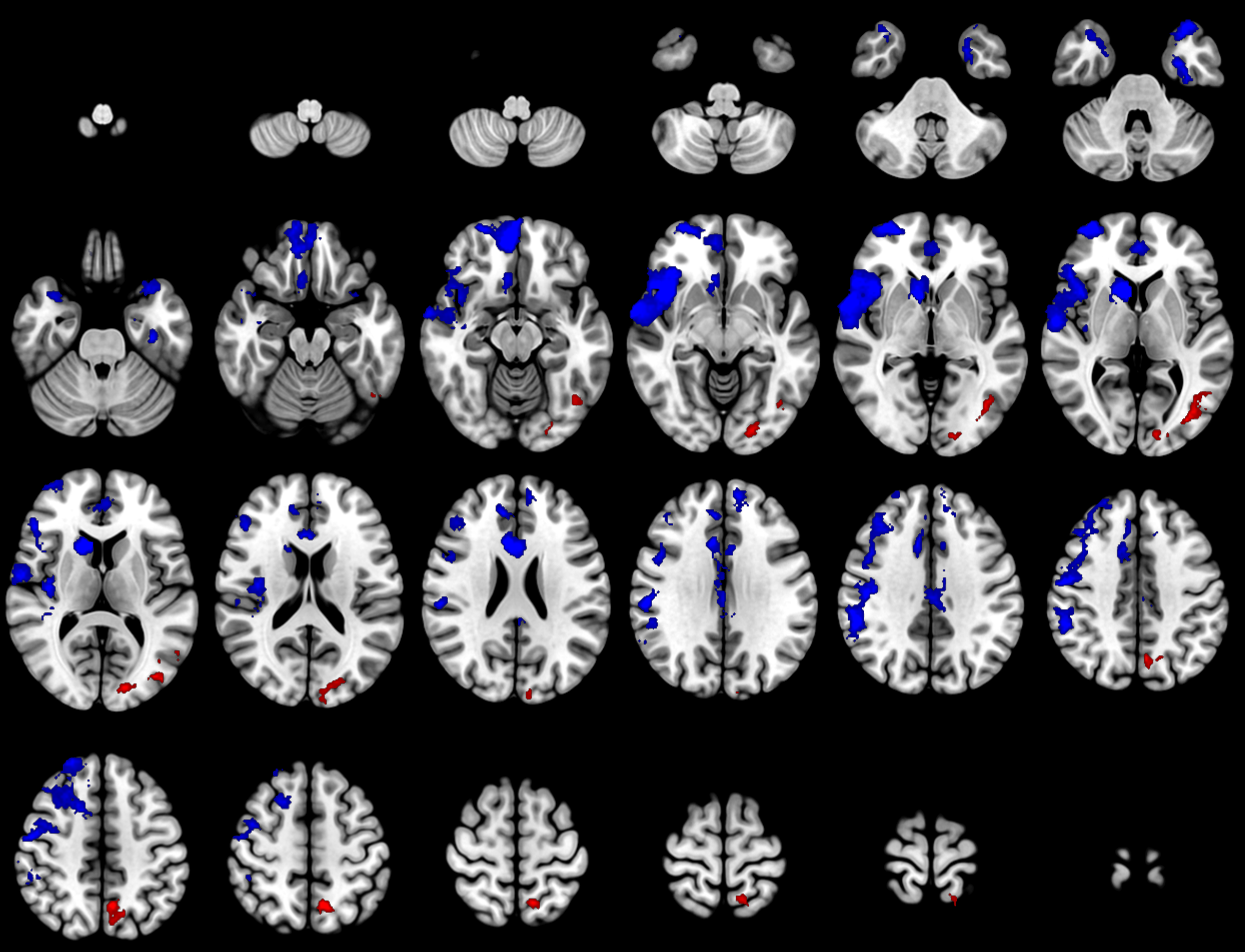  **(B)** |

**Supplementary Figure 3.** (A) SPECT images of Patient 3. (B) Higher (red) or lower (blue) regional cerebral blood flow of Patient 3 compared to the control group. Images are shown in radiological convention.

| 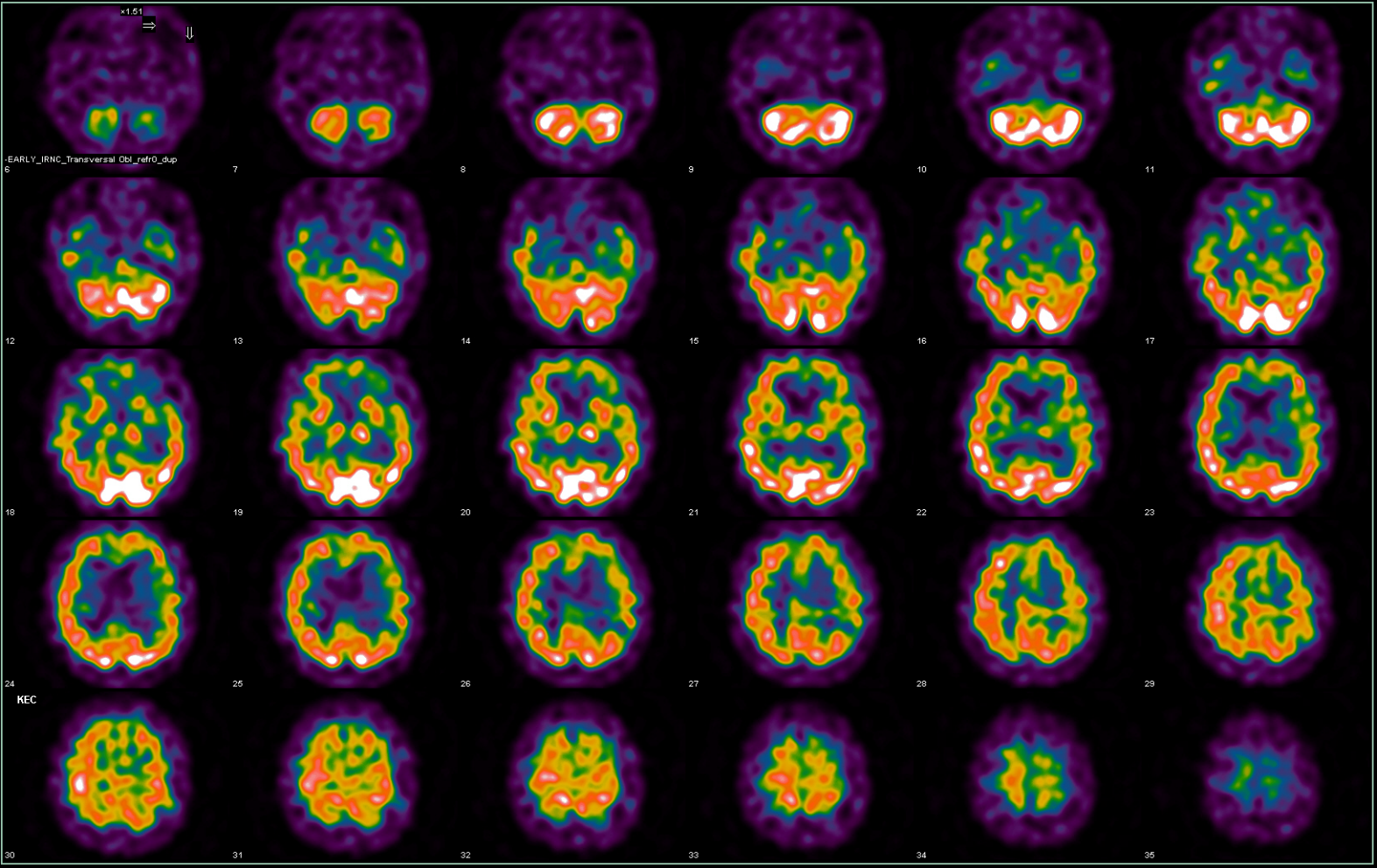  **(A)** |
| --- |
| 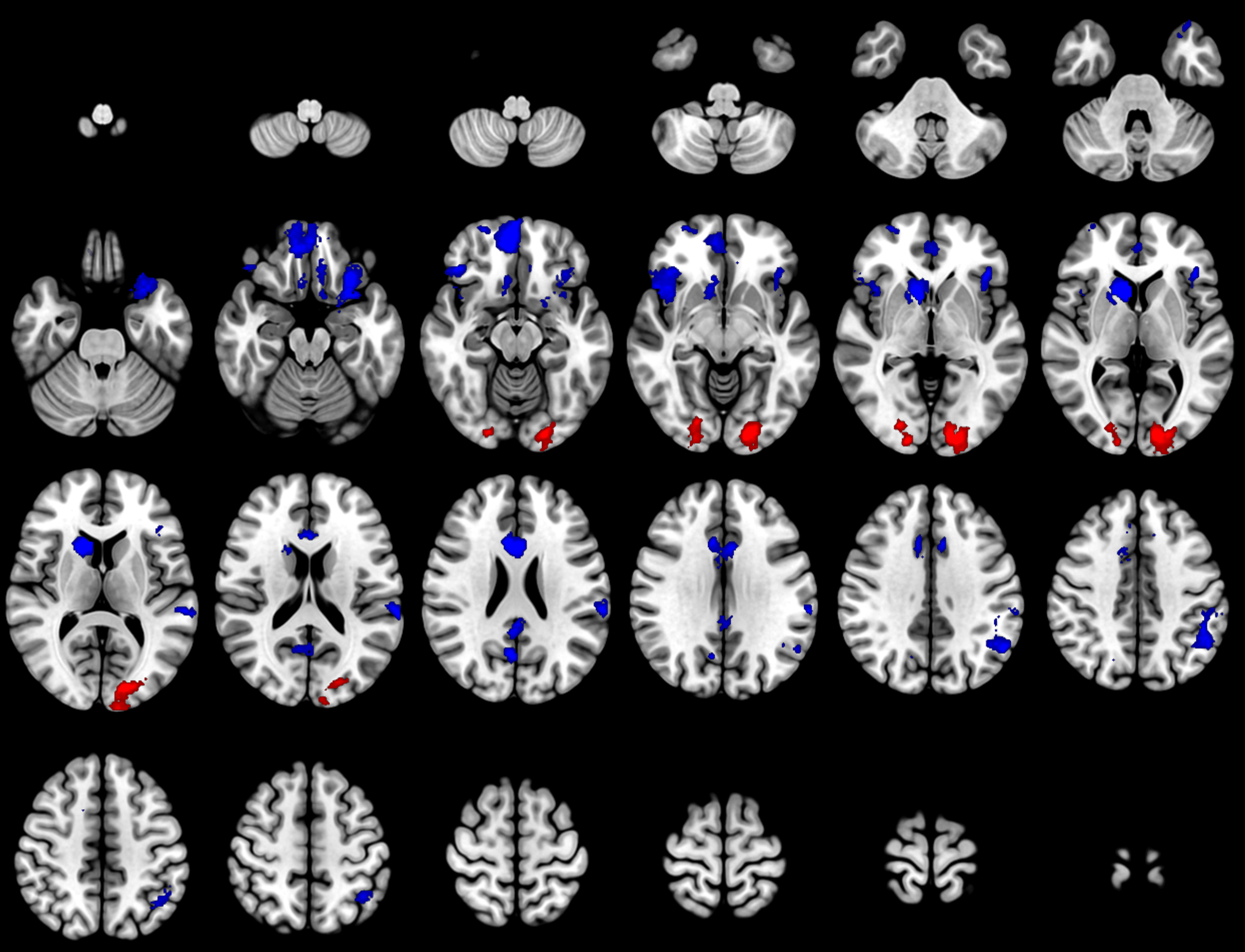 |

**Supplementary Figure 4.** (A) SPECT images of Patient 4. (B) Higher (red) or lower (blue) regional cerebral blood flow of Patient 4 compared to the control group. Images are shown in radiological convention.

**(B)**
